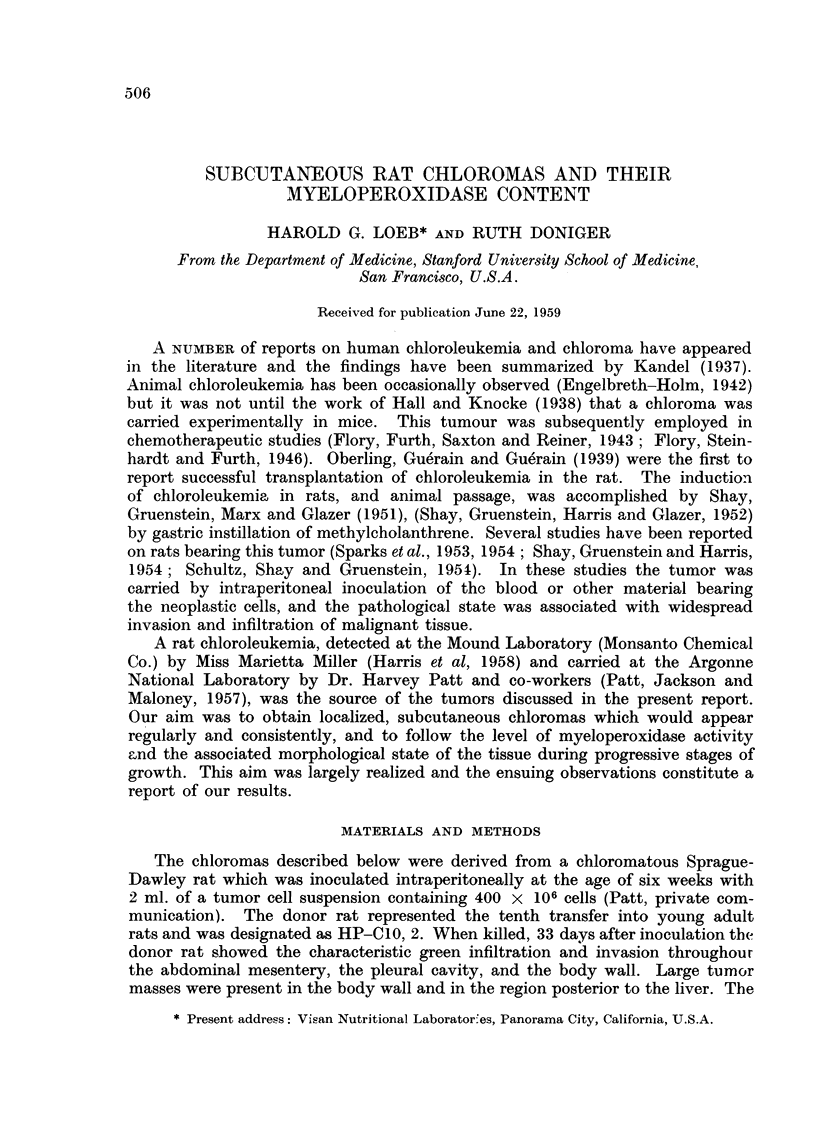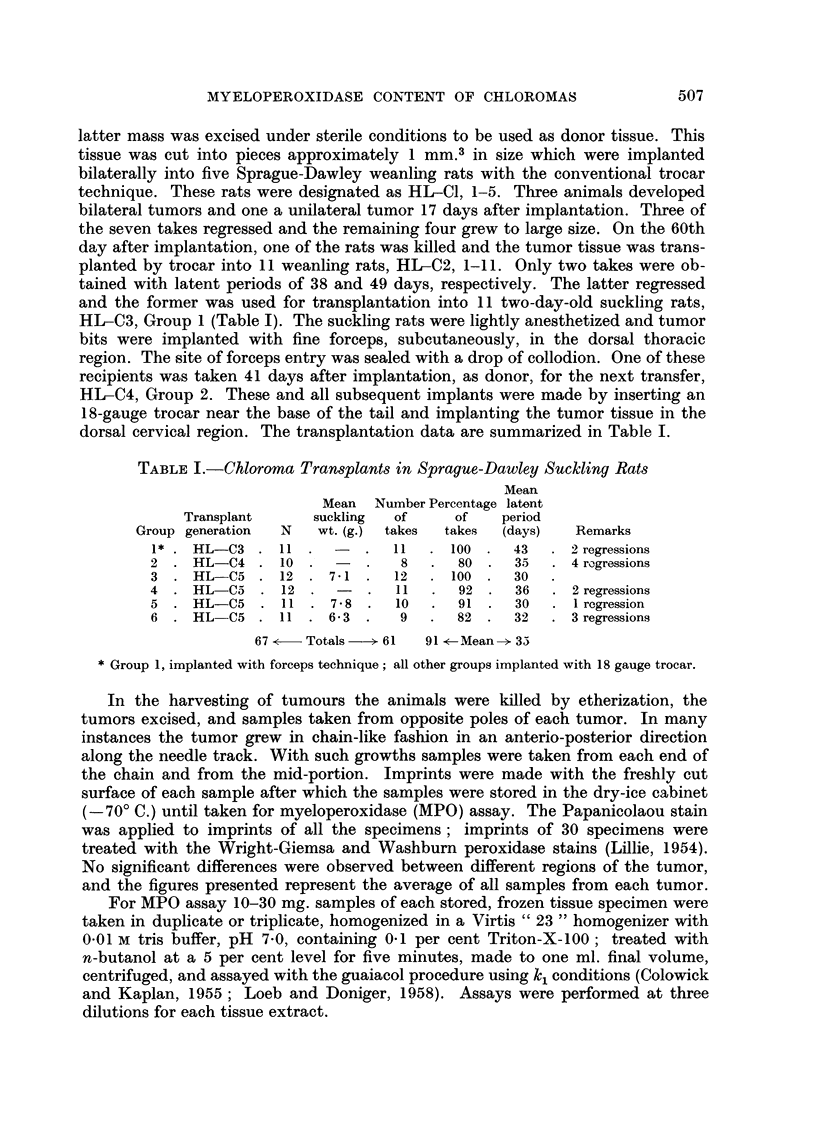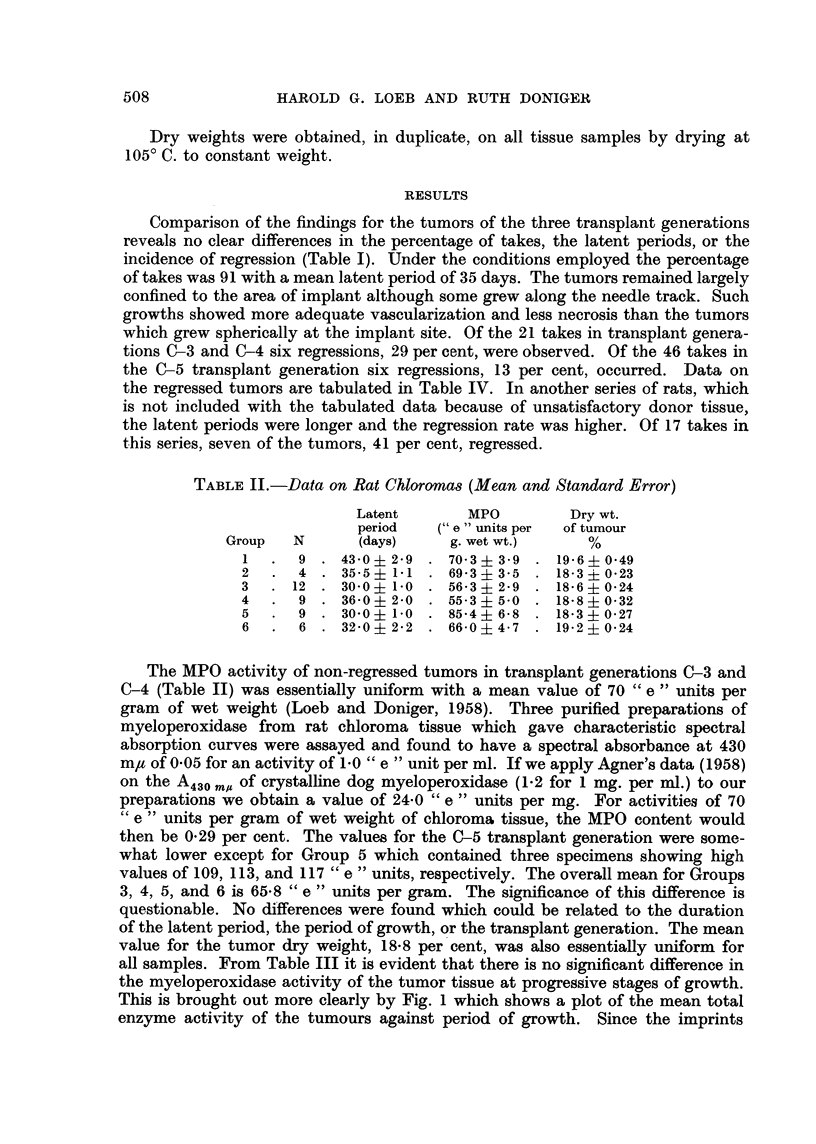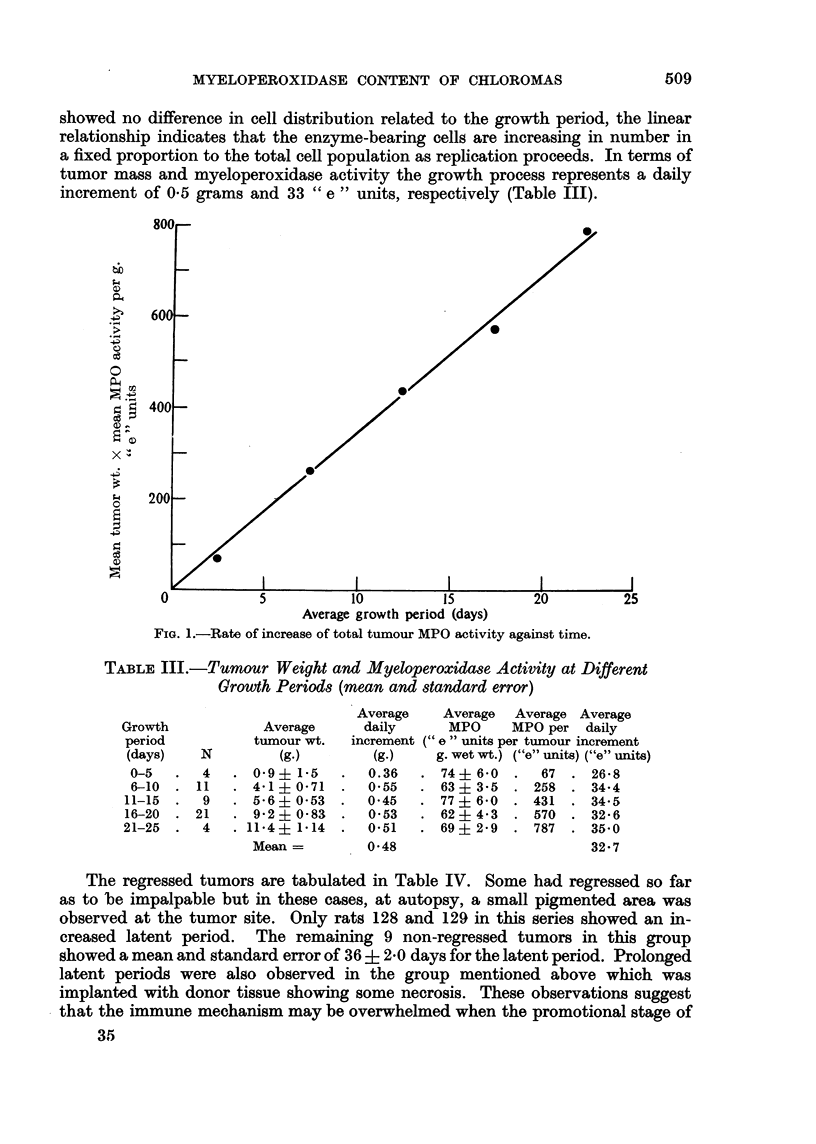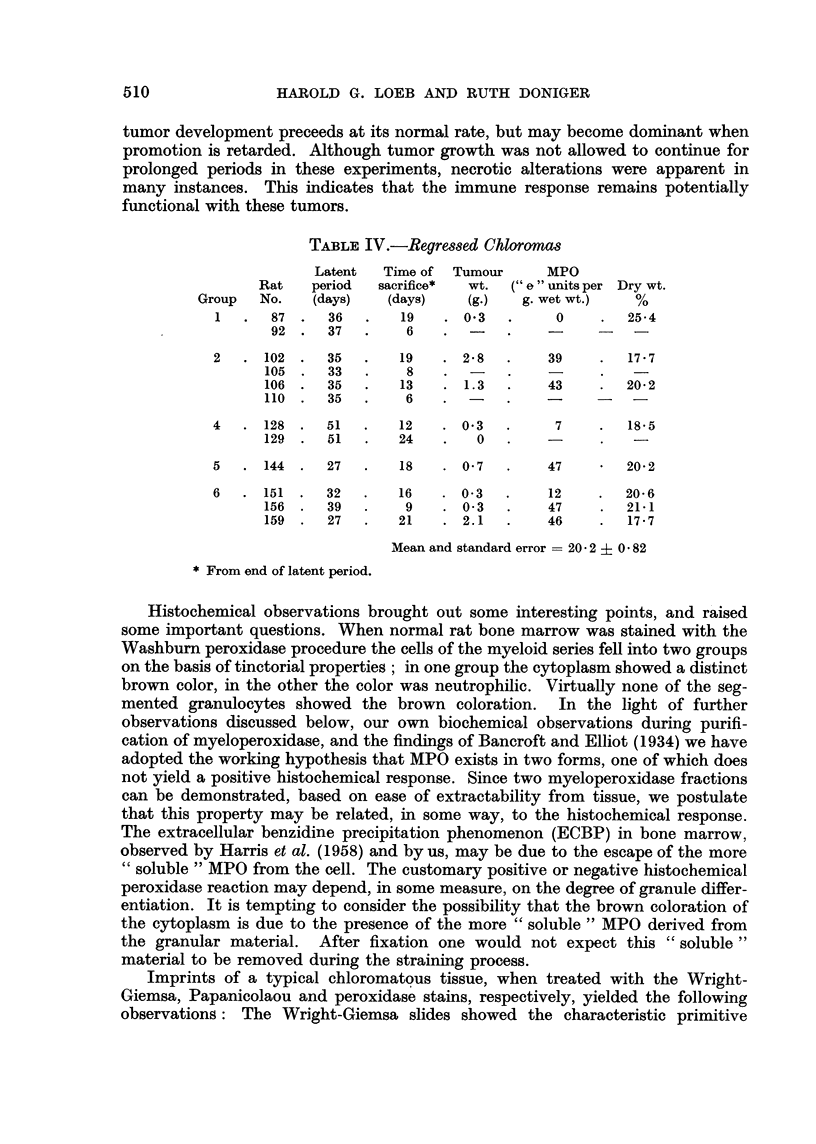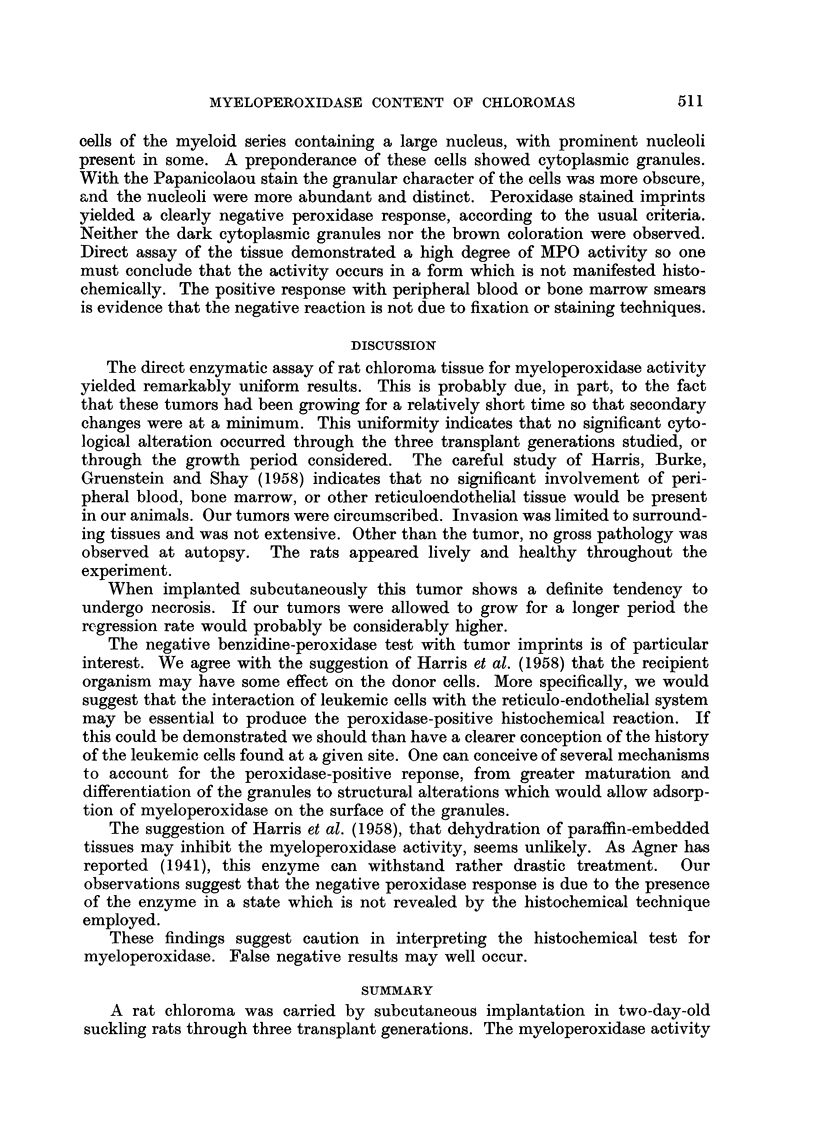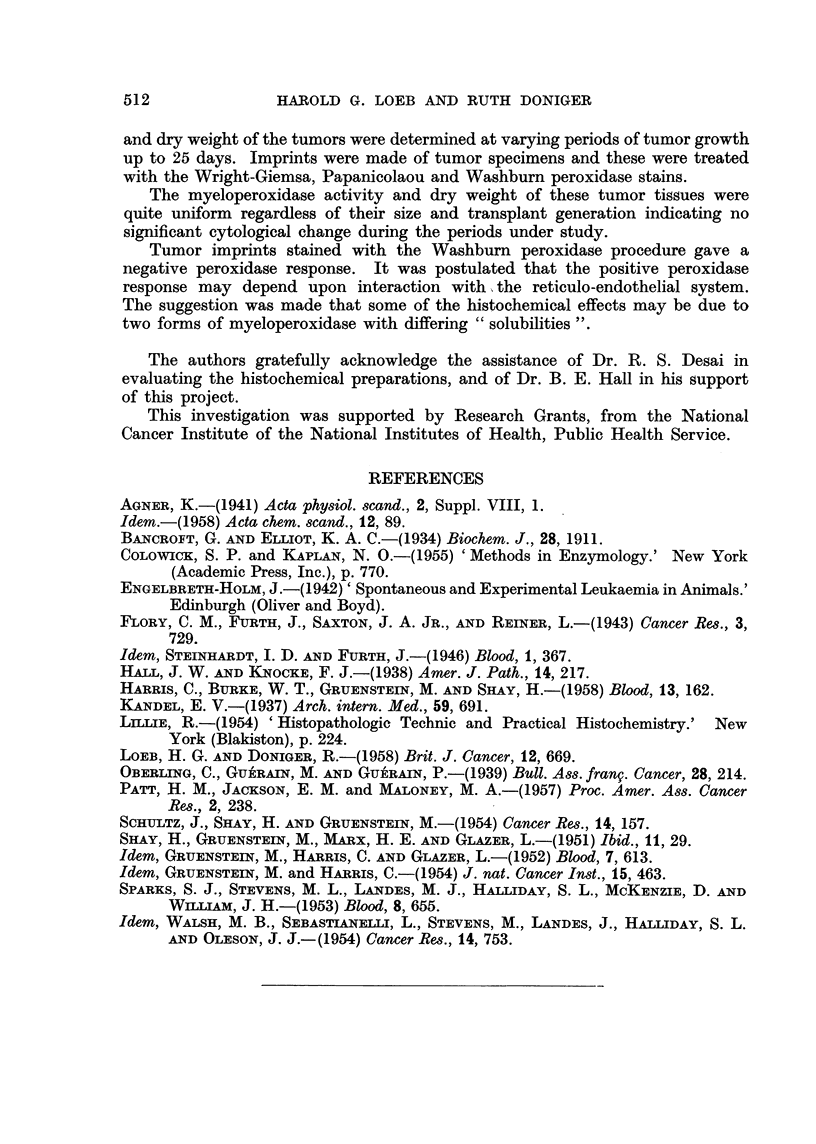# Subcutaneous Rat Chloromas and their Myeloperoxidase Content

**DOI:** 10.1038/bjc.1959.56

**Published:** 1959-09

**Authors:** Harold G. Loeb, Ruth Doniger


### SUBCUTANEOUS RAT CHLOROMAS AND THEIR MYELOPEROXIDASE CONTENT

HAROLD G. LOEB* AND RUTH DONIGER

*From the Department of Medicine, Stanford University School of Medicine, San Francisco, U.S.A.*

Received for publication June 22, 1959

A NUMBER of reports on human chloroleukemia and chloroma have appeared in the literature and the findings have been summarized by Kandel (1937). Animal chloroleukemia has been occasionally observed (Engelbreth–Holm, 1942) but it was not until the work of Hall and Knocke (1938) that a chloroma was carried experimentally in mice. This tumour was subsequently employed in chemotherapeutic studies (Flory, Furth, Saxton and Reiner, 1943 ; Flory, Steinhardt and Furth, 1946). Oberling, Guérain and Guérain (1939) were the first to report successful transplantation of chloroleukemia in the rat. The induction of chloroleukemia in rats, and animal passage, was accomplished by Shay, Gruenstein, Marx and Glazer (1951), (Shay, Gruenstein, Harris and Glazer, 1952) by gastric instillation of methylcholanthrene. Several studies have been reported on rats bearing this tumor (Sparks *et al.*, 1953, 1954 ; Shay, Gruenstein and Harris, 1954 ; Schultz, Shay and Gruenstein, 1954). In these studies the tumor was carried by intraperitoneal inoculation of the blood or other material bearing the neoplastic cells, and the pathological state was associated with widespread invasion and infiltration of malignant tissue.

A rat chloroleukemia, detected at the Mound Laboratory (Monsanto Chemical Co.) by Miss Marietta Miller (Harris *et al*, 1958) and carried at the Argonne National Laboratory by Dr. Harvey Patt and co-workers (Patt, Jackson and Maloney, 1957), was the source of the tumors discussed in the present report. Our aim was to obtain localized, subcutaneous chloromas which would appear regularly and consistently, and to follow the level of myeloperoxidase activity and the associated morphological state of the tissue during progressive stages of growth. This aim was largely realized and the ensuing observations constitute a report of our results.

### MATERIALS AND METHODS

The chloromas described below were derived from a chloromatous Sprague-Dawley rat which was inoculated intraperitoneally at the age of six weeks with 2 ml. of a tumor cell suspension containing $400 \times 10^6$ cells (Patt, private communication). The donor rat represented the tenth transfer into young adult rats and was designated as HP–C10, 2. When killed, 33 days after inoculation the donor rat showed the characteristic green infiltration and invasion throughout the abdominal mesentery, the pleural cavity, and the body wall. Large tumor masses were present in the body wall and in the region posterior to the liver. The

* Present address : Visan Nutritional Laboratories, Panorama City, California, U.S.A.

latter mass was excised under sterile conditions to be used as donor tissue. This tissue was cut into pieces approximately 1 mm.[3] in size which were implanted bilaterally into five Sprague-Dawley weanling rats with the conventional trocar technique. These rats were designated as HL–Cl, 1–5. Three animals developed bilateral tumors and one a unilateral tumor 17 days after implantation. Three of the seven takes regressed and the remaining four grew to large size. On the 60th day after implantation, one of the rats was killed and the tumor tissue was transplanted by trocar into 11 weanling rats, HL–C2, 1–11. Only two takes were obtained with latent periods of 38 and 49 days, respectively. The latter regressed and the former was used for transplantation into 11 two-day-old suckling rats, HL–C3, Group 1 (Table I). The suckling rats were lightly anesthetized and tumor bits were implanted with fine forceps, subcutaneously, in the dorsal thoracic region. The site of forceps entry was sealed with a drop of collodion. One of these recipients was taken 41 days after implantation, as donor, for the next transfer, HL–C4, Group 2. These and all subsequent implants were made by inserting an 18-gauge trocar near the base of the tail and implanting the tumor tissue in the dorsal cervical region. The transplantation data are summarized in Table I.

TABLE I.—*Chloroma Transplants in Sprague-Dawley Suckling Rats*

| Group | Transplant generation | N | Mean suckling wt. (g.) | Number of takes | Percentage of takes | Mean latent period (days) | Remarks |
|---|---|---|---|---|---|---|---|
| 1* | HL—C3 | 11 | — | 11 | 100 | 43 | 2 regressions |
| 2 | HL—C4 | 10 | — | 8 | 80 | 35 | 4 regressions |
| 3 | HL—C5 | 12 | 7·1 | 12 | 100 | 30 | |
| 4 | HL—C5 | 12 | — | 11 | 92 | 36 | 2 regressions |
| 5 | HL—C5 | 11 | 7·8 | 10 | 91 | 30 | 1 regression |
| 6 | HL—C5 | 11 | 6·3 | 9 | 82 | 32 | 3 regressions |
| | | 67 ← | Totals | → 61 | 91 ← | Mean → 35 | |

* Group 1, implanted with forceps technique ; all other groups implanted with 18 gauge trocar.

In the harvesting of tumours the animals were killed by etherization, the tumors excised, and samples taken from opposite poles of each tumor. In many instances the tumor grew in chain-like fashion in an anterio-posterior direction along the needle track. With such growths samples were taken from each end of the chain and from the mid-portion. Imprints were made with the freshly cut surface of each sample after which the samples were stored in the dry-ice cabinet (−70° C.) until taken for myeloperoxidase (MPO) assay. The Papanicolaou stain was applied to imprints of all the specimens ; imprints of 30 specimens were treated with the Wright-Giemsa and Washburn peroxidase stains (Lillie, 1954). No significant differences were observed between different regions of the tumor, and the figures presented represent the average of all samples from each tumor.

For MPO assay 10–30 mg. samples of each stored, frozen tissue specimen were taken in duplicate or triplicate, homogenized in a Virtis " 23 " homogenizer with 0·01 M tris buffer, pH 7·0, containing 0·1 per cent Triton-X-100 ; treated with *n*-butanol at a 5 per cent level for five minutes, made to one ml. final volume, centrifuged, and assayed with the guaiacol procedure using $k_1$ conditions (Colowick and Kaplan, 1955 ; Loeb and Doniger, 1958). Assays were performed at three dilutions for each tissue extract.

Dry weights were obtained, in duplicate, on all tissue samples by drying at 105° C. to constant weight.

### RESULTS

Comparison of the findings for the tumors of the three transplant generations reveals no clear differences in the percentage of takes, the latent periods, or the incidence of regression (Table I). Under the conditions employed the percentage of takes was 91 with a mean latent period of 35 days. The tumors remained largely confined to the area of implant although some grew along the needle track. Such growths showed more adequate vascularization and less necrosis than the tumors which grew spherically at the implant site. Of the 21 takes in transplant generations C–3 and C–4 six regressions, 29 per cent, were observed. Of the 46 takes in the C–5 transplant generation six regressions, 13 per cent, occurred. Data on the regressed tumors are tabulated in Table IV. In another series of rats, which is not included with the tabulated data because of unsatisfactory donor tissue, the latent periods were longer and the regression rate was higher. Of 17 takes in this series, seven of the tumors, 41 per cent, regressed.

TABLE II.—*Data on Rat Chloromas (Mean and Standard Error)*

| Group | N | Latent period (days) | MPO (" e " units per g. wet wt.) | Dry wt. of tumour % |
|---|---|---|---|---|
| 1 | 9 | 43·0 ± 2·9 | 70·3 ± 3·9 | 19·6 ± 0·49 |
| 2 | 4 | 35·5 ± 1·1 | 69·3 ± 3·5 | 18·3 ± 0·23 |
| 3 | 12 | 30·0 ± 1·0 | 56·3 ± 2·9 | 18·6 ± 0·24 |
| 4 | 9 | 36·0 ± 2·0 | 55·3 ± 5·0 | 18·8 ± 0·32 |
| 5 | 9 | 30·0 ± 1·0 | 85·4 ± 6·8 | 18·3 ± 0·27 |
| 6 | 6 | 32·0 ± 2·2 | 66·0 ± 4·7 | 19·2 ± 0·24 |

The MPO activity of non-regressed tumors in transplant generations C–3 and C–4 (Table II) was essentially uniform with a mean value of 70 " e " units per gram of wet weight (Loeb and Doniger, 1958). Three purified preparations of myeloperoxidase from rat chloroma tissue which gave characteristic spectral absorption curves were assayed and found to have a spectral absorbance at 430 m$\mu$ of 0·05 for an activity of 1·0 " e " unit per ml. If we apply Agner's data (1958) on the $A_{430\ m\mu}$ of crystalline dog myeloperoxidase (1·2 for 1 mg. per ml.) to our preparations we obtain a value of 24·0 " e " units per mg. For activities of 70 " e " units per gram of wet weight of chloroma tissue, the MPO content would then be 0·29 per cent. The values for the C–5 transplant generation were somewhat lower except for Group 5 which contained three specimens showing high values of 109, 113, and 117 " e " units, respectively. The overall mean for Groups 3, 4, 5, and 6 is 65·8 " e " units per gram. The significance of this difference is questionable. No differences were found which could be related to the duration of the latent period, the period of growth, or the transplant generation. The mean value for the tumor dry weight, 18·8 per cent, was also essentially uniform for all samples. From Table III it is evident that there is no significant difference in the myeloperoxidase activity of the tumor tissue at progressive stages of growth. This is brought out more clearly by Fig. 1 which shows a plot of the mean total enzyme activity of the tumours against period of growth. Since the imprints showed no difference in cell distribution related to the growth period, the linear relationship indicates that the enzyme-bearing cells are increasing in number in a fixed proportion to the total cell population as replication proceeds. In terms of tumor mass and myeloperoxidase activity the growth process represents a daily increment of 0·5 grams and 33 " e " units, respectively (Table III).

FIG. 1.—Rate of increase of total tumour MPO activity against time.

TABLE III.—*Tumour Weight and Myeloperoxidase Activity at Different Growth Periods (mean and standard error)*

| Growth period (days) | N | Average tumour wt. (g.) | Average daily increment (g.) | Average MPO (" e " units per g. wet wt.) | Average MPO per tumour ("e" units) | Average daily increment ("e" units) |
|---|---|---|---|---|---|---|
| 0–5 | 4 | 0·9 ± 1·5 | 0.36 | 74 ± 6·0 | 67 | 26·8 |
| 6–10 | 11 | 4·1 ± 0·71 | 0·55 | 63 ± 3·5 | 258 | 34·4 |
| 11–15 | 9 | 5·6 ± 0·53 | 0·45 | 77 ± 6·0 | 431 | 34·5 |
| 16–20 | 21 | 9·2 ± 0·83 | 0·53 | 62 ± 4·3 | 570 | 32·6 |
| 21–25 | 4 | 11·4 ± 1·14 | 0·51 | 69 ± 2·9 | 787 | 35·0 |
| | | Mean = | 0·48 | | | 32·7 |

The regressed tumors are tabulated in Table IV. Some had regressed so far as to be impalpable but in these cases, at autopsy, a small pigmented area was observed at the tumor site. Only rats 128 and 129 in this series showed an increased latent period. The remaining 9 non-regressed tumors in this group showed a mean and standard error of 36 ± 2·0 days for the latent period. Prolonged latent periods were also observed in the group mentioned above which was implanted with donor tissue showing some necrosis. These observations suggest that the immune mechanism may be overwhelmed when the promotional stage of tumor development preceeds at its normal rate, but may become dominant when promotion is retarded. Although tumor growth was not allowed to continue for prolonged periods in these experiments, necrotic alterations were apparent in many instances. This indicates that the immune response remains potentially functional with these tumors.

TABLE IV.—*Regressed Chloromas*

| Group | Rat No. | Latent period (days) | Time of sacrifice* (days) | Tumour wt. (g.) | MPO ("e" units per g. wet wt.) | Dry wt. % |
|---|---|---|---|---|---|---|
| 1 | 87 | 36 | 19 | 0·3 | 0 | 25·4 |
| | 92 | 37 | 6 | — | — | — |
| 2 | 102 | 35 | 19 | 2·8 | 39 | 17·7 |
| | 105 | 33 | 8 | — | — | — |
| | 106 | 35 | 13 | 1.3 | 43 | 20·2 |
| | 110 | 35 | 6 | — | — | — |
| 4 | 128 | 51 | 12 | 0·3 | 7 | 18·5 |
| | 129 | 51 | 24 | 0 | — | — |
| 5 | 144 | 27 | 18 | 0·7 | 47 | 20·2 |
| 6 | 151 | 32 | 16 | 0·3 | 12 | 20·6 |
| | 156 | 39 | 9 | 0·3 | 47 | 21·1 |
| | 159 | 27 | 21 | 2.1 | 46 | 17·7 |

Mean and standard error = $20 \cdot 2 \pm 0 \cdot 82$

* From end of latent period.

Histochemical observations brought out some interesting points, and raised some important questions. When normal rat bone marrow was stained with the Washburn peroxidase procedure the cells of the myeloid series fell into two groups on the basis of tinctorial properties ; in one group the cytoplasm showed a distinct brown color, in the other the color was neutrophilic. Virtually none of the segmented granulocytes showed the brown coloration. In the light of further observations discussed below, our own biochemical observations during purification of myeloperoxidase, and the findings of Bancroft and Elliot (1934) we have adopted the working hypothesis that MPO exists in two forms, one of which does not yield a positive histochemical response. Since two myeloperoxidase fractions can be demonstrated, based on ease of extractability from tissue, we postulate that this property may be related, in some way, to the histochemical response. The extracellular benzidine precipitation phenomenon (ECBP) in bone marrow, observed by Harris *et al.* (1958) and by us, may be due to the escape of the more " soluble " MPO from the cell. The customary positive or negative histochemical peroxidase reaction may depend, in some measure, on the degree of granule differentiation. It is tempting to consider the possibility that the brown coloration of the cytoplasm is due to the presence of the more " soluble " MPO derived from the granular material. After fixation one would not expect this " soluble " material to be removed during the straining process.

Imprints of a typical chloromatous tissue, when treated with the Wright-Giemsa, Papanicolaou and peroxidase stains, respectively, yielded the following observations : The Wright-Giemsa slides showed the characteristic primitive cells of the myeloid series containing a large nucleus, with prominent nucleoli present in some. A preponderance of these cells showed cytoplasmic granules. With the Papanicolaou stain the granular character of the cells was more obscure, and the nucleoli were more abundant and distinct. Peroxidase stained imprints yielded a clearly negative peroxidase response, according to the usual criteria. Neither the dark cytoplasmic granules nor the brown coloration were observed. Direct assay of the tissue demonstrated a high degree of MPO activity so one must conclude that the activity occurs in a form which is not manifested histochemically. The positive response with peripheral blood or bone marrow smears is evidence that the negative reaction is not due to fixation or staining techniques.

### DISCUSSION

The direct enzymatic assay of rat chloroma tissue for myeloperoxidase activity yielded remarkably uniform results. This is probably due, in part, to the fact that these tumors had been growing for a relatively short time so that secondary changes were at a minimum. This uniformity indicates that no significant cytological alteration occurred through the three transplant generations studied, or through the growth period considered. The careful study of Harris, Burke, Gruenstein and Shay (1958) indicates that no significant involvement of peripheral blood, bone marrow, or other reticuloendothelial tissue would be present in our animals. Our tumors were circumscribed. Invasion was limited to surrounding tissues and was not extensive. Other than the tumor, no gross pathology was observed at autopsy. The rats appeared lively and healthy throughout the experiment.

When implanted subcutaneously this tumor shows a definite tendency to undergo necrosis. If our tumors were allowed to grow for a longer period the regression rate would probably be considerably higher.

The negative benzidine-peroxidase test with tumor imprints is of particular interest. We agree with the suggestion of Harris *et al.* (1958) that the recipient organism may have some effect on the donor cells. More specifically, we would suggest that the interaction of leukemic cells with the reticulo-endothelial system may be essential to produce the peroxidase-positive histochemical reaction. If this could be demonstrated we should than have a clearer conception of the history of the leukemic cells found at a given site. One can conceive of several mechanisms to account for the peroxidase-positive reponse, from greater maturation and differentiation of the granules to structural alterations which would allow adsorption of myeloperoxidase on the surface of the granules.

The suggestion of Harris *et al.* (1958), that dehydration of paraffin-embedded tissues may inhibit the myeloperoxidase activity, seems unlikely. As Agner has reported (1941), this enzyme can withstand rather drastic treatment. Our observations suggest that the negative peroxidase response is due to the presence of the enzyme in a state which is not revealed by the histochemical technique employed.

These findings suggest caution in interpreting the histochemical test for myeloperoxidase. False negative results may well occur.

### SUMMARY

A rat chloroma was carried by subcutaneous implantation in two-day-old suckling rats through three transplant generations. The myeloperoxidase activity and dry weight of the tumors were determined at varying periods of tumor growth up to 25 days. Imprints were made of tumor specimens and these were treated with the Wright-Giemsa, Papanicolaou and Washburn peroxidase stains.

The myeloperoxidase activity and dry weight of these tumor tissues were quite uniform regardless of their size and transplant generation indicating no significant cytological change during the periods under study.

Tumor imprints stained with the Washburn peroxidase procedure gave a negative peroxidase response. It was postulated that the positive peroxidase response may depend upon interaction with the reticulo-endothelial system. The suggestion was made that some of the histochemical effects may be due to two forms of myeloperoxidase with differing " solubilities ".

The authors gratefully acknowledge the assistance of Dr. R. S. Desai in evaluating the histochemical preparations, and of Dr. B. E. Hall in his support of this project.

This investigation was supported by Research Grants, from the National Cancer Institute of the National Institutes of Health, Public Health Service.

### REFERENCES

AGNER, K.—(1941) *Acta physiol. scand.*, **2**, Suppl. VIII, 1.

*Idem.*—(1958) *Acta chem. scand.*, **12**, 89.

BANCROFT, G. AND ELLIOT, K. A. C.—(1934) *Biochem. J.*, **28**, 1911.

COLOWICK, S. P. and KAPLAN, N. O.—(1955) 'Methods in Enzymology.' New York (Academic Press, Inc.), p. 770.

ENGELBRETH-HOLM, J.—(1942) 'Spontaneous and Experimental Leukaemia in Animals.' Edinburgh (Oliver and Boyd).

FLORY, C. M., FURTH, J., SAXTON, J. A. JR., AND REINER, L.—(1943) *Cancer Res.*, **3**, 729.

*Idem*, STEINHARDT, I. D. AND FURTH, J.—(1946) *Blood*, **1**, 367.

HALL, J. W. AND KNOCKE, F. J.—(1938) *Amer. J. Path.*, **14**, 217.

HARRIS, C., BURKE, W. T., GRUENSTEIN, M. AND SHAY, H.—(1958) *Blood*, **13**, 162.

KANDEL, E. V.—(1937) *Arch. intern. Med.*, **59**, 691.

LILLIE, R.—(1954) 'Histopathologic Technic and Practical Histochemistry.' New York (Blakiston), p. 224.

LOEB, H. G. AND DONIGER, R.—(1958) *Brit. J. Cancer*, **12**, 669.

OBERLING, C., GUÉRAIN, M. AND GUÉRAIN, P.—(1939) *Bull. Ass. franç. Cancer*, **28**, 214.

PATT, H. M., JACKSON, E. M. and MALONEY, M. A.—(1957) *Proc. Amer. Ass. Cancer Res.*, **2**, 238.

SCHULTZ, J., SHAY, H. AND GRUENSTEIN, M.—(1954) *Cancer Res.*, **14**, 157.

SHAY, H., GRUENSTEIN, M., MARX, H. E. AND GLAZER, L.—(1951) *Ibid.*, **11**, 29.

*Idem*, GRUENSTEIN, M., HARRIS, C. AND GLAZER, L.—(1952) *Blood*, **7**, 613.

*Idem*, GRUENSTEIN, M. and HARRIS, C.—(1954) *J. nat. Cancer Inst.*, **15**, 463.

SPARKS, S. J., STEVENS, M. L., LANDES, M. J., HALLIDAY, S. L., MCKENZIE, D. AND WILLIAM, J. H.—(1953) *Blood*, **8**, 655.

*Idem*, WALSH, M. B., SEBASTIANELLI, L., STEVENS, M., LANDES, J., HALLIDAY, S. L. AND OLESON, J. J.—(1954) *Cancer Res.*, **14**, 753.